# Supplementary figures and images for: Tenotomy-induced muscle atrophy is sex-specific and independent of NFκB
Source: eLife. 2022 Dec 12;11:e82016. doi: 10.7554/eLife.82016 (PMC9873255; doi:10.7554/eLife.82016)

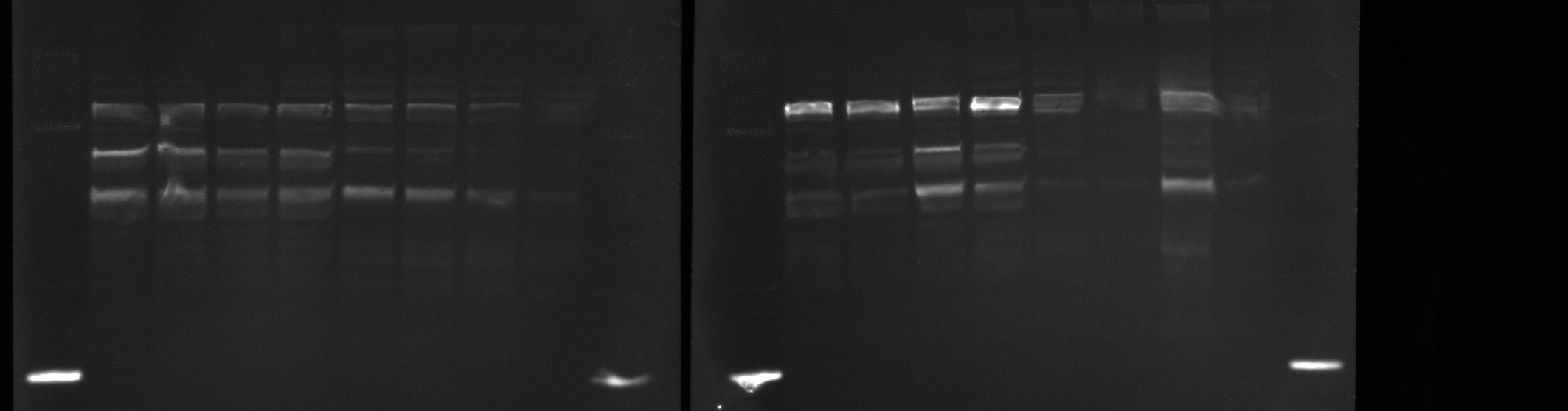

Supplement: Figure 2—source data 2. [file elife-82016-fig2-data2.zip › Fig 2 - source data 2/Fig 2E_p50_p65_800.TIF]

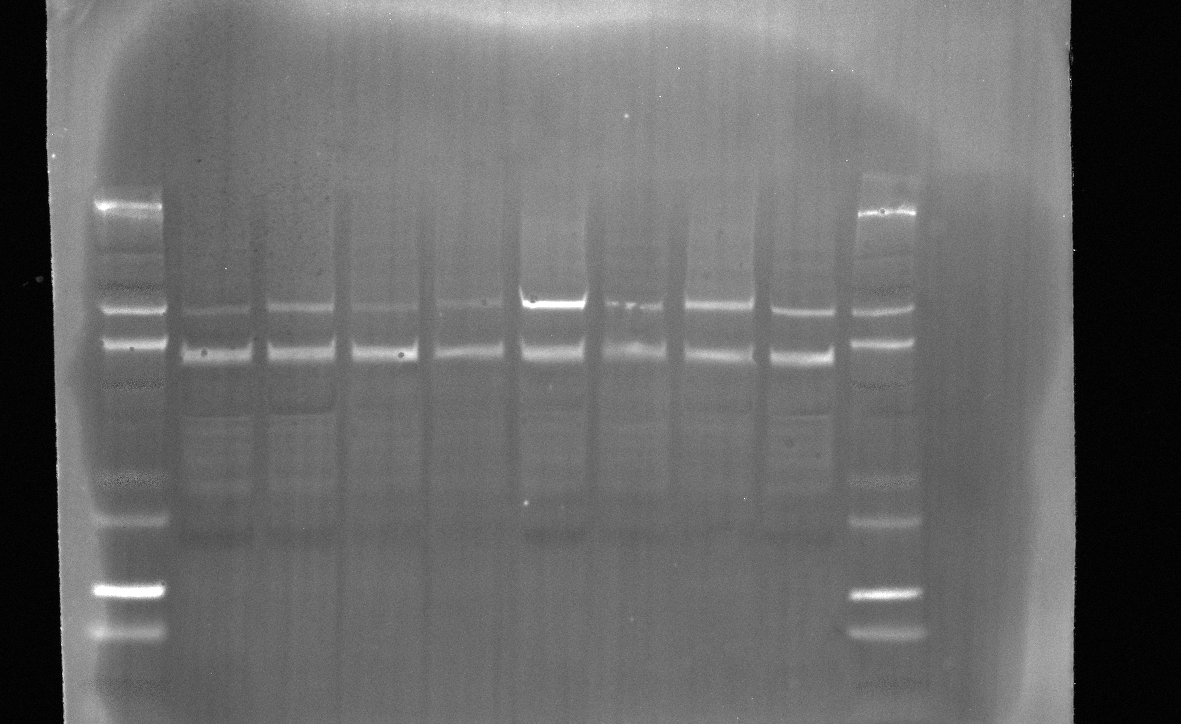

Supplement: Figure 2—source data 2. [file elife-82016-fig2-data2.zip › Fig 2 - source data 2/Fig2D_IKKb_Akt_800.TIF]

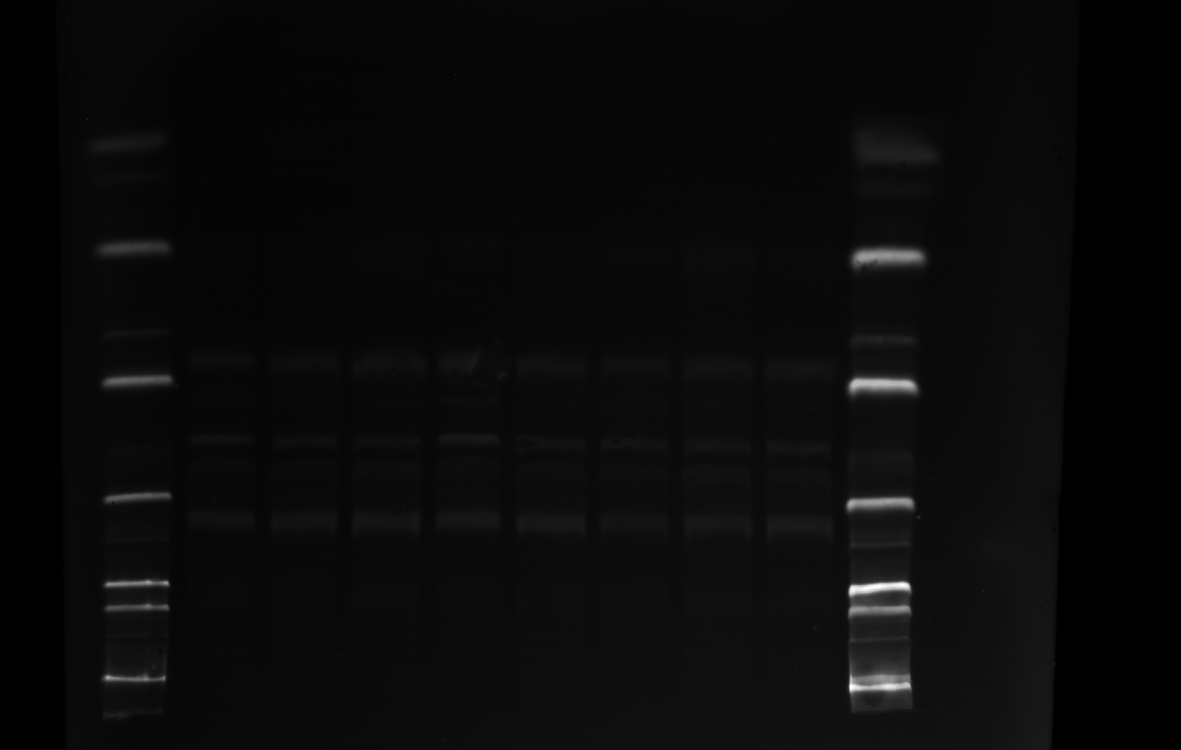

Supplement: Figure 2—source data 2. [file elife-82016-fig2-data2.zip › Fig 2 - source data 2/Fig2C_IKKb_Akt_Actin_700.TIF]

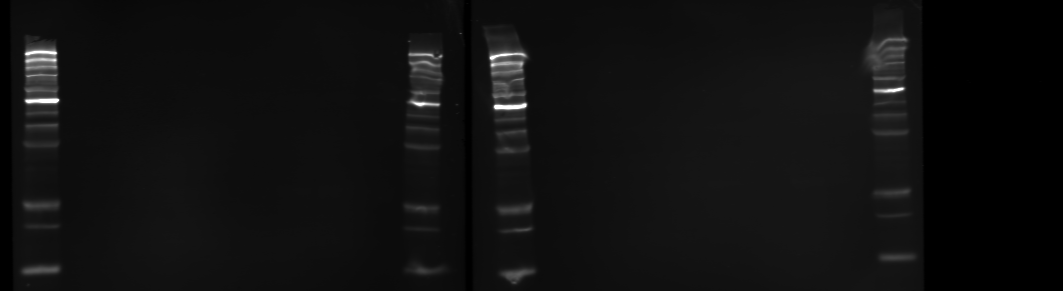

Supplement: Figure 2—source data 2. [file elife-82016-fig2-data2.zip › Fig 2 - source data 2/Fig 2E_HistoneH3_GAPDH_700.TIF]

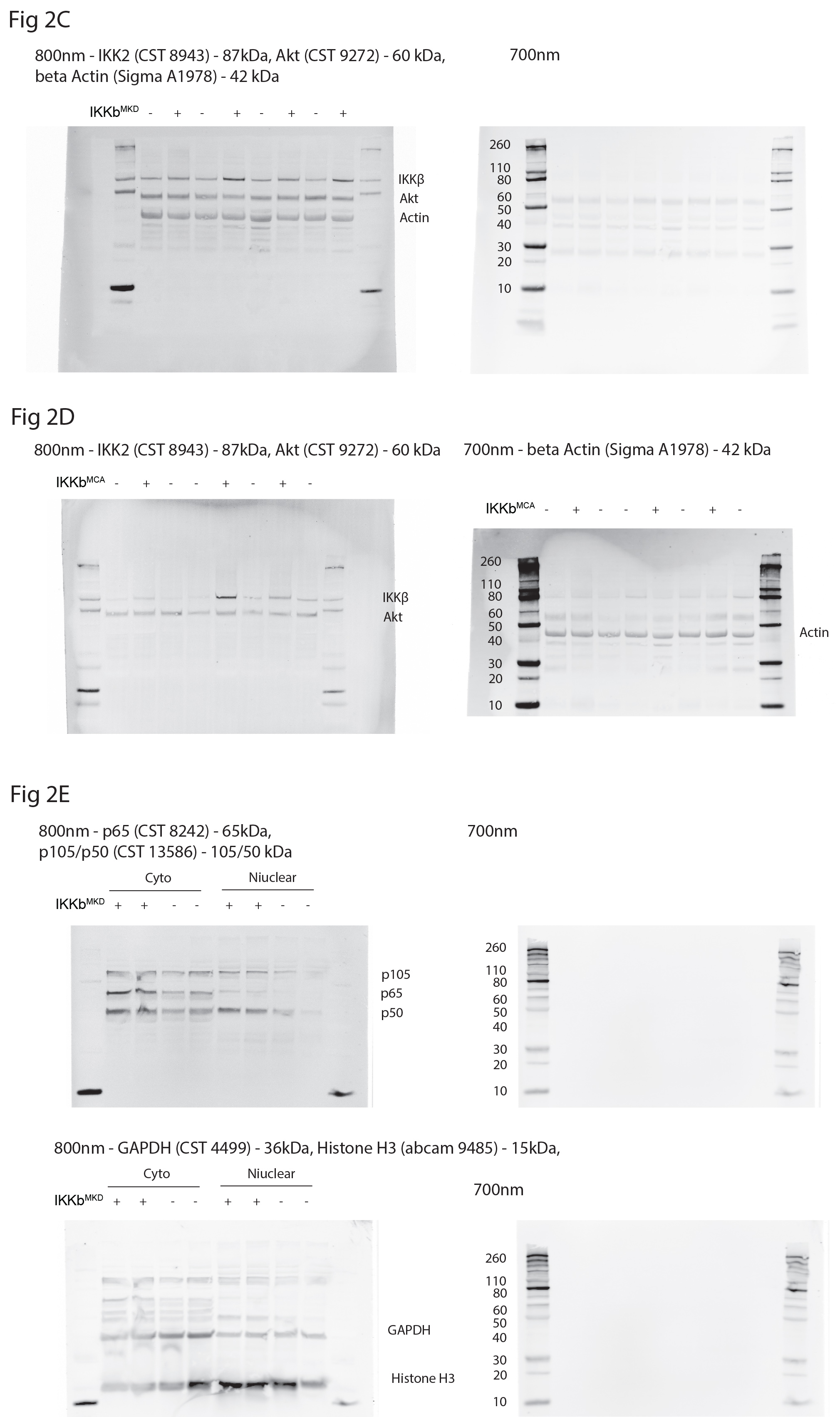

Supplement: Figure 2—source data 2. [file elife-82016-fig2-data2.zip › Fig 2 - source data 2/Fig2_Blots.jpg]

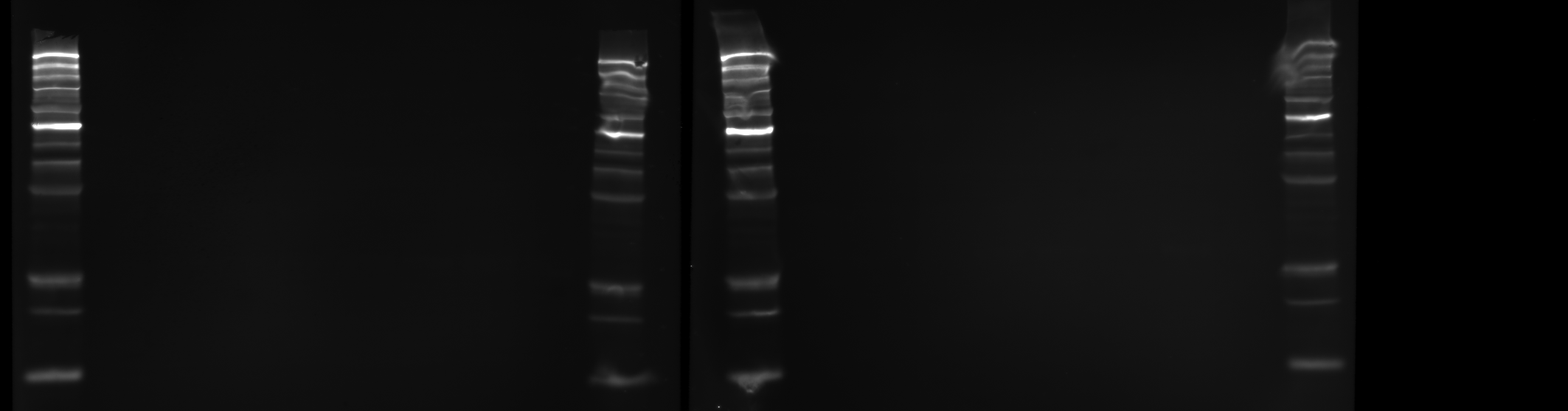

Supplement: Figure 2—source data 2. [file elife-82016-fig2-data2.zip › Fig 2 - source data 2/Fig 2E_p50_p65_700.TIF]

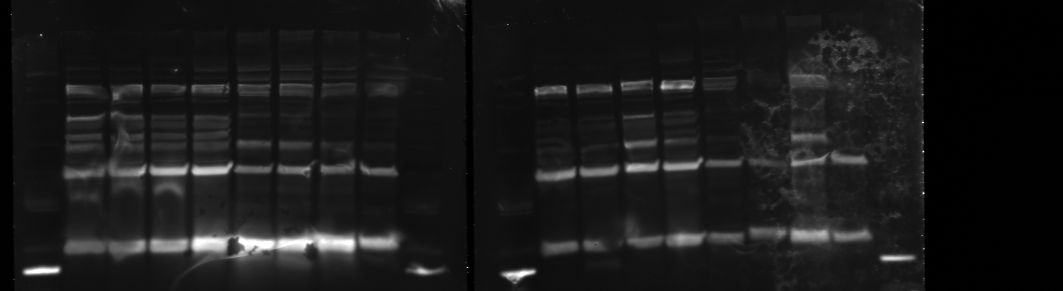

Supplement: Figure 2—source data 2. [file elife-82016-fig2-data2.zip › Fig 2 - source data 2/Fig 2E_HistoneH3_GAPDH_800.TIF]

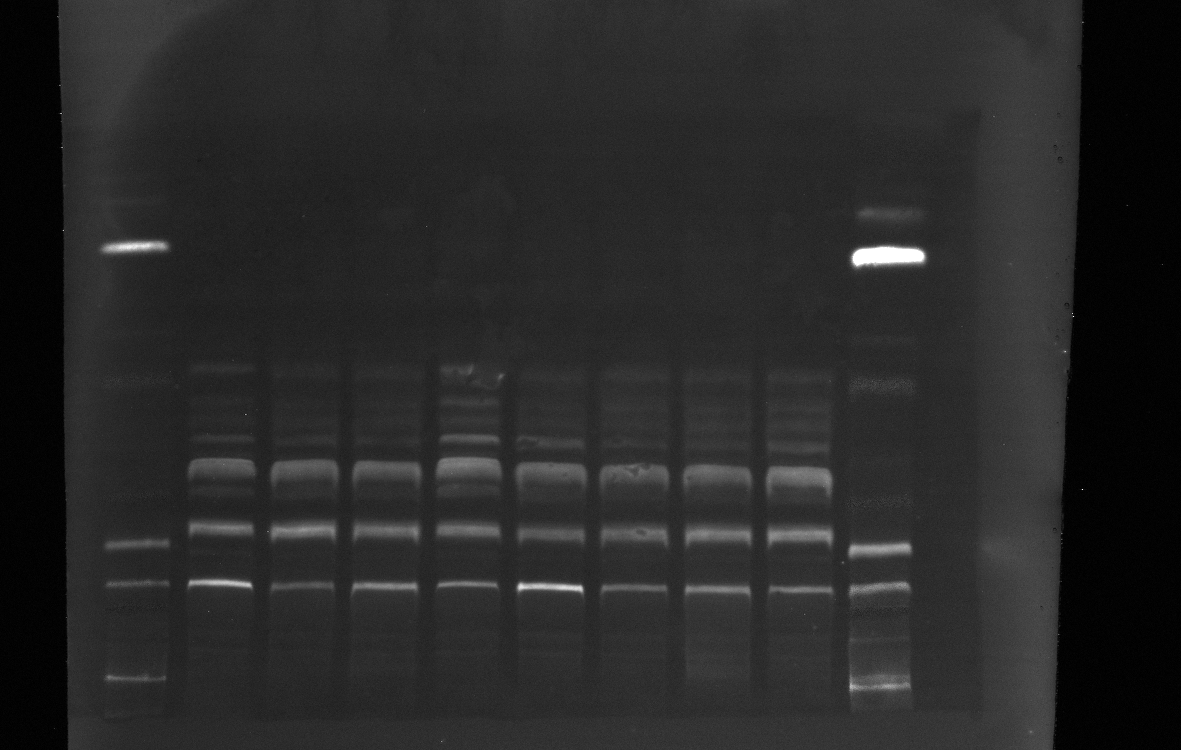

Supplement: Figure 2—source data 2. [file elife-82016-fig2-data2.zip › Fig 2 - source data 2/Fig2C_IKKb_Akt_Actin_800.TIF]

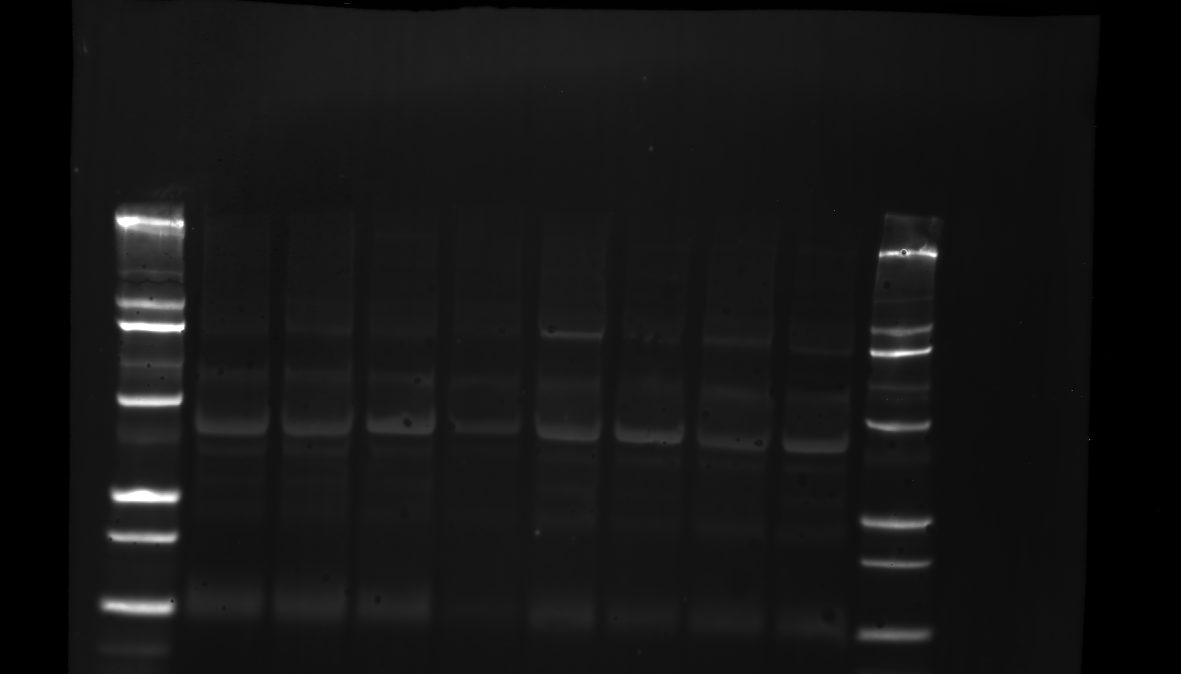

Supplement: Figure 2—source data 2. [file elife-82016-fig2-data2.zip › Fig 2 - source data 2/Fig2D_Actin_700.TIF]

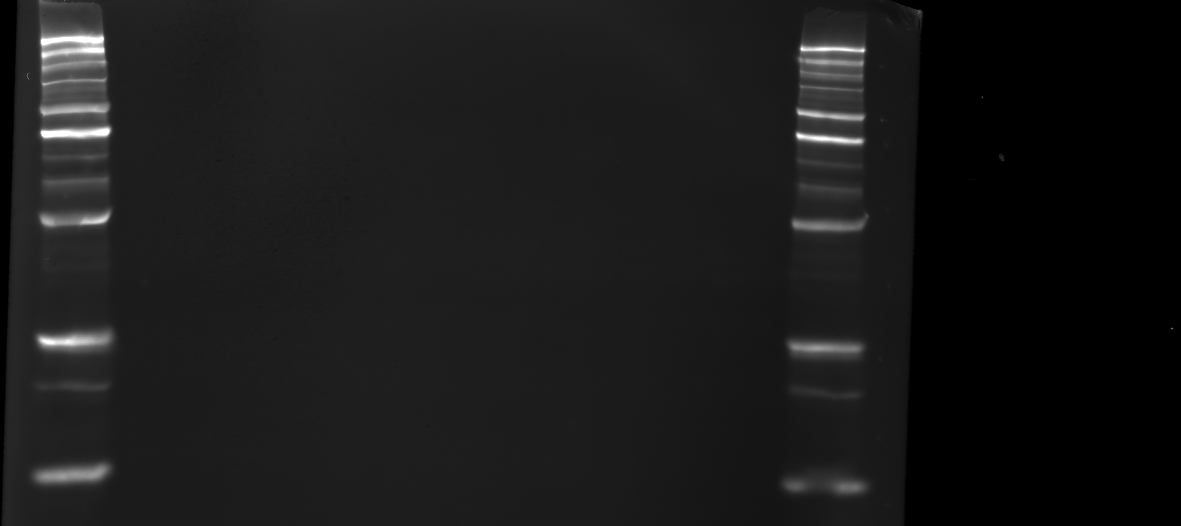

Supplement: Figure 2—figure supplement 1—source data 2. [file elife-82016-fig2-figsupp1-data2.zip › Fig 2S1 - source data 2/Fig 2S1_GAPDH_700.TIF]

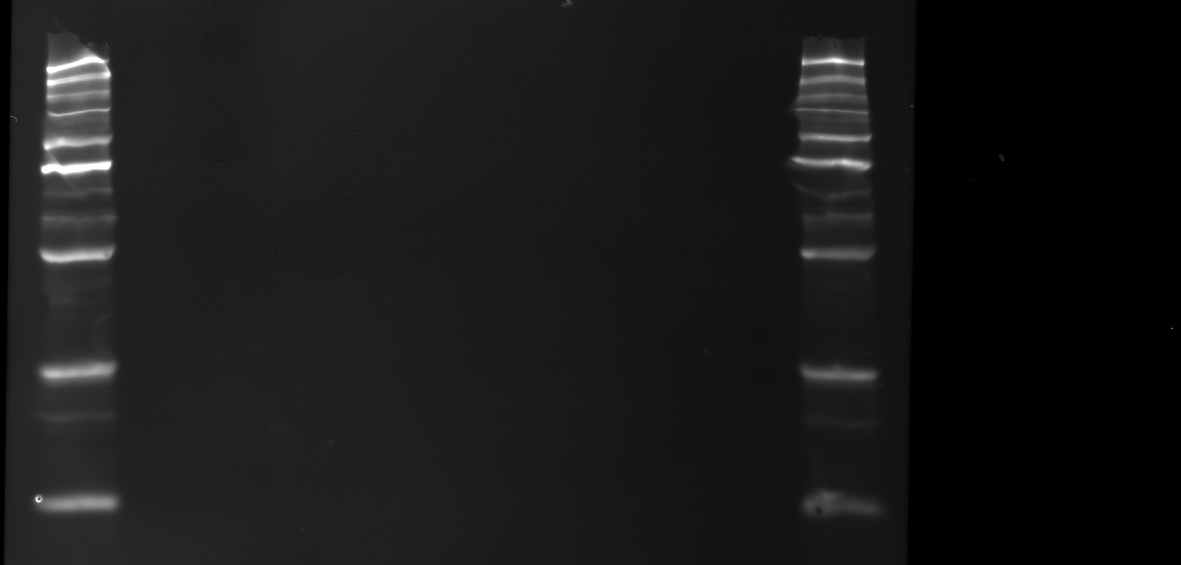

Supplement: Figure 2—figure supplement 1—source data 2. [file elife-82016-fig2-figsupp1-data2.zip › Fig 2S1 - source data 2/Fig 2S1_HistoneH3_700.TIF]

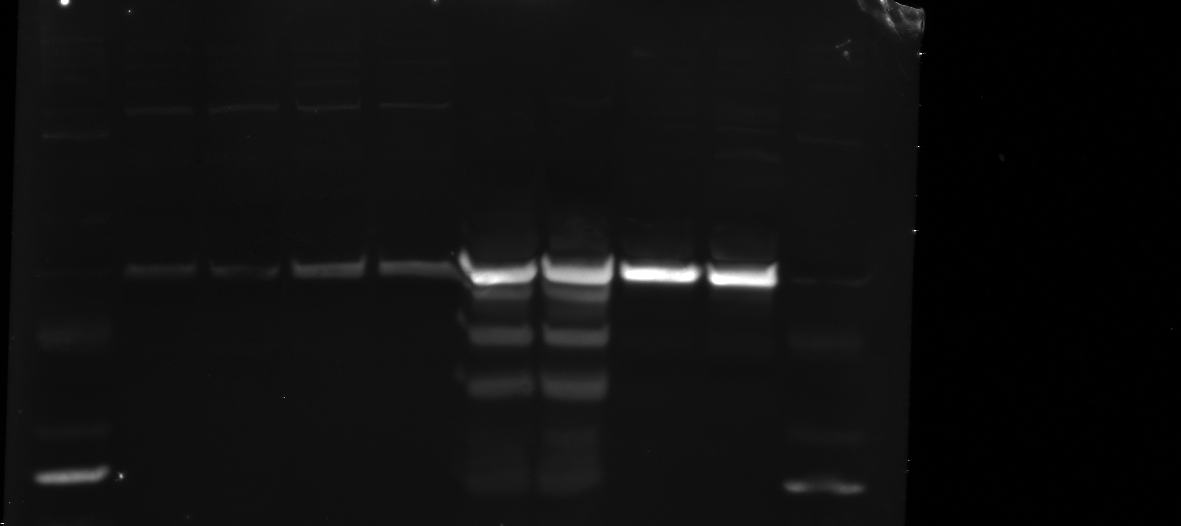

Supplement: Figure 2—figure supplement 1—source data 2. [file elife-82016-fig2-figsupp1-data2.zip › Fig 2S1 - source data 2/Fig 2S1_GAPDH_800.TIF]

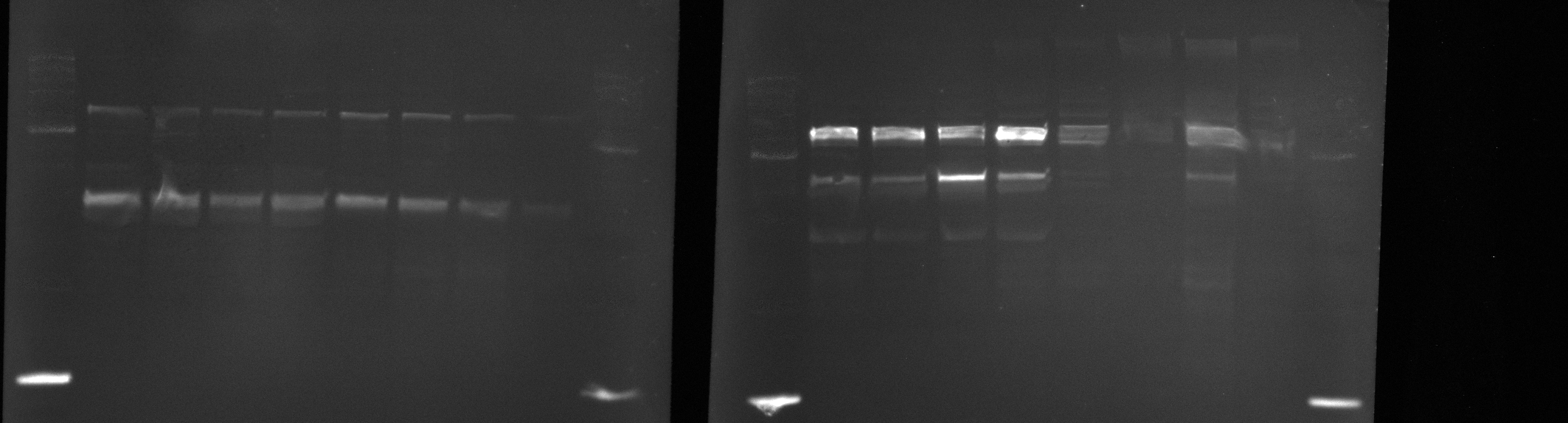

Supplement: Figure 2—figure supplement 1—source data 2. [file elife-82016-fig2-figsupp1-data2.zip › Fig 2S1 - source data 2/Fig 2S1_p50p65_800.TIF]

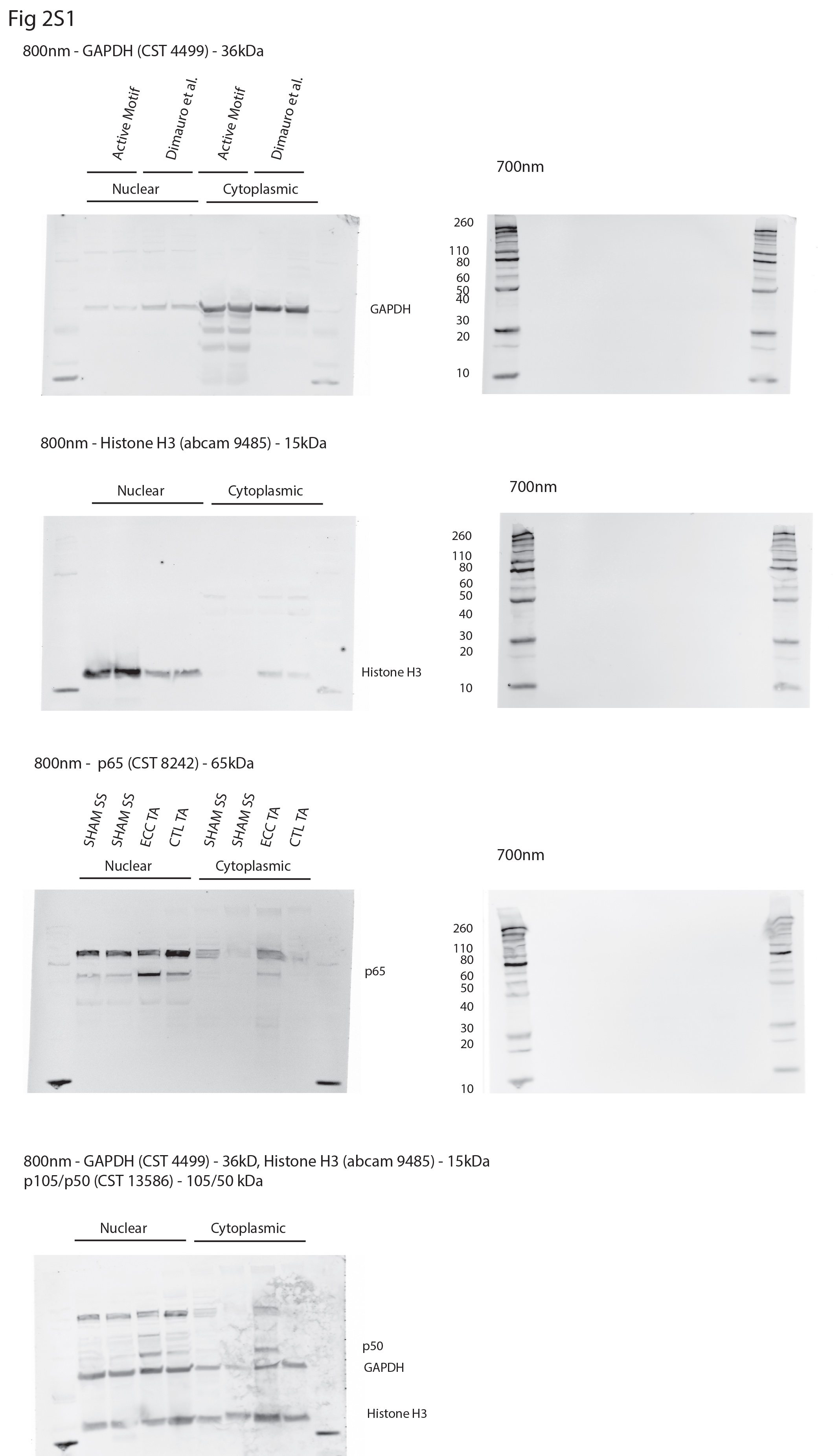

Supplement: Figure 2—figure supplement 1—source data 2. [file elife-82016-fig2-figsupp1-data2.zip › Fig 2S1 - source data 2/Fig2S1_Blots.jpg]

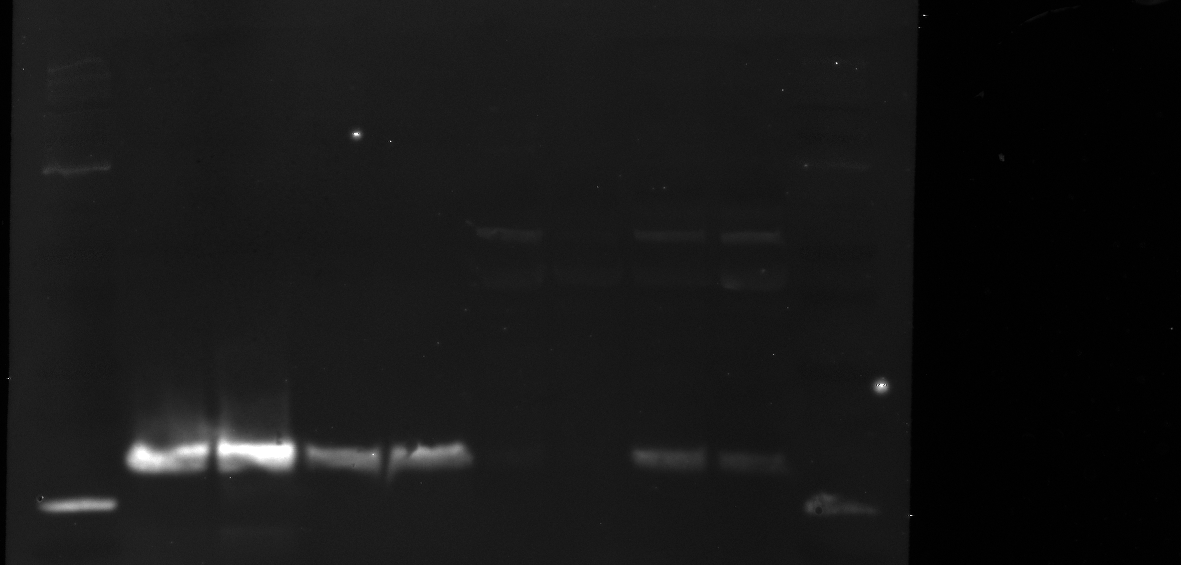

Supplement: Figure 2—figure supplement 1—source data 2. [file elife-82016-fig2-figsupp1-data2.zip › Fig 2S1 - source data 2/Fig 2S1_HistoneH3_800.TIF]

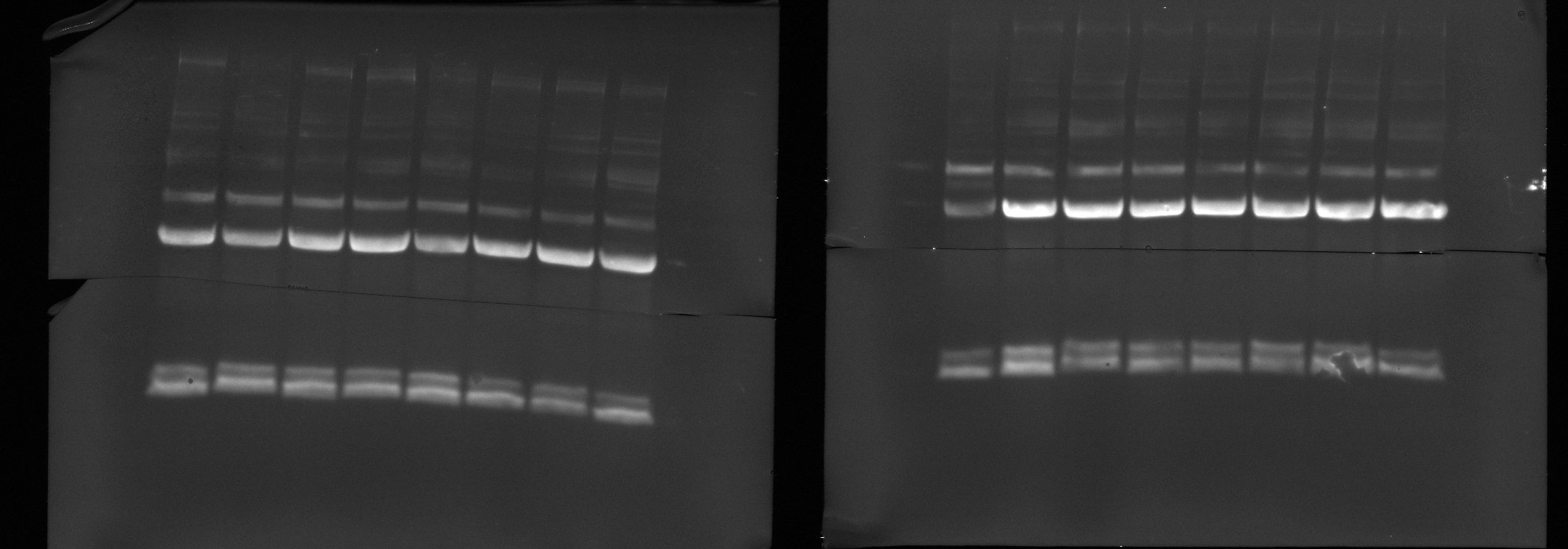

Supplement: Figure 7—source data 2. [file elife-82016-fig7-data2.zip › Fig 7 - source data 2/Fig7D2_p62_LC3_Actin_800.TIF]

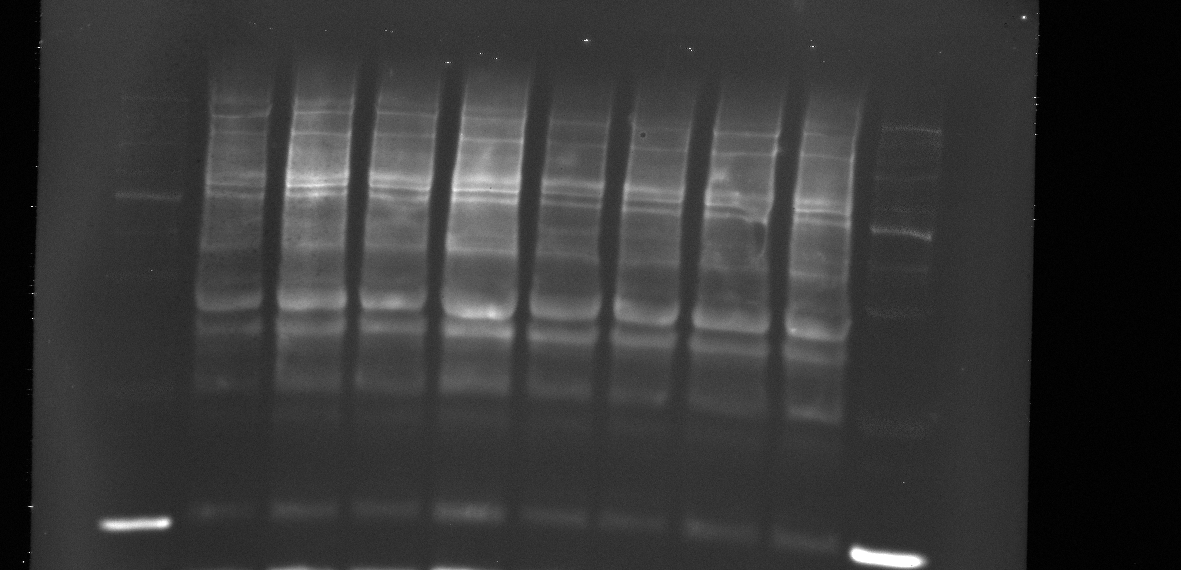

Supplement: Figure 7—source data 2. [file elife-82016-fig7-data2.zip › Fig 7 - source data 2/Fig 7B_Ubiquitin_800.TIF]

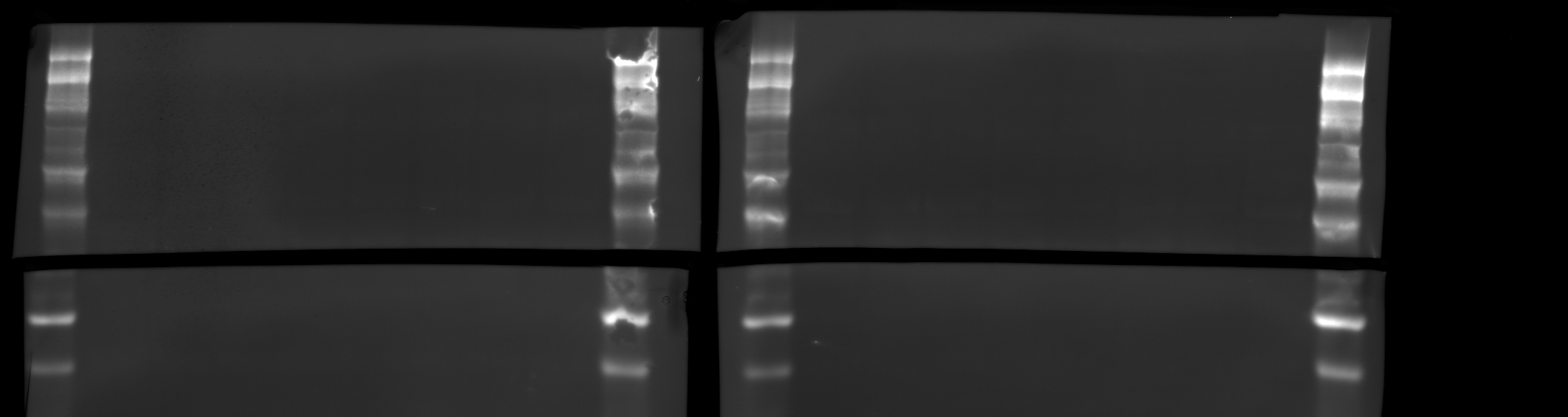

Supplement: Figure 7—source data 2. [file elife-82016-fig7-data2.zip › Fig 7 - source data 2/Fig7D_p62_LC3_Actin_700.TIF]

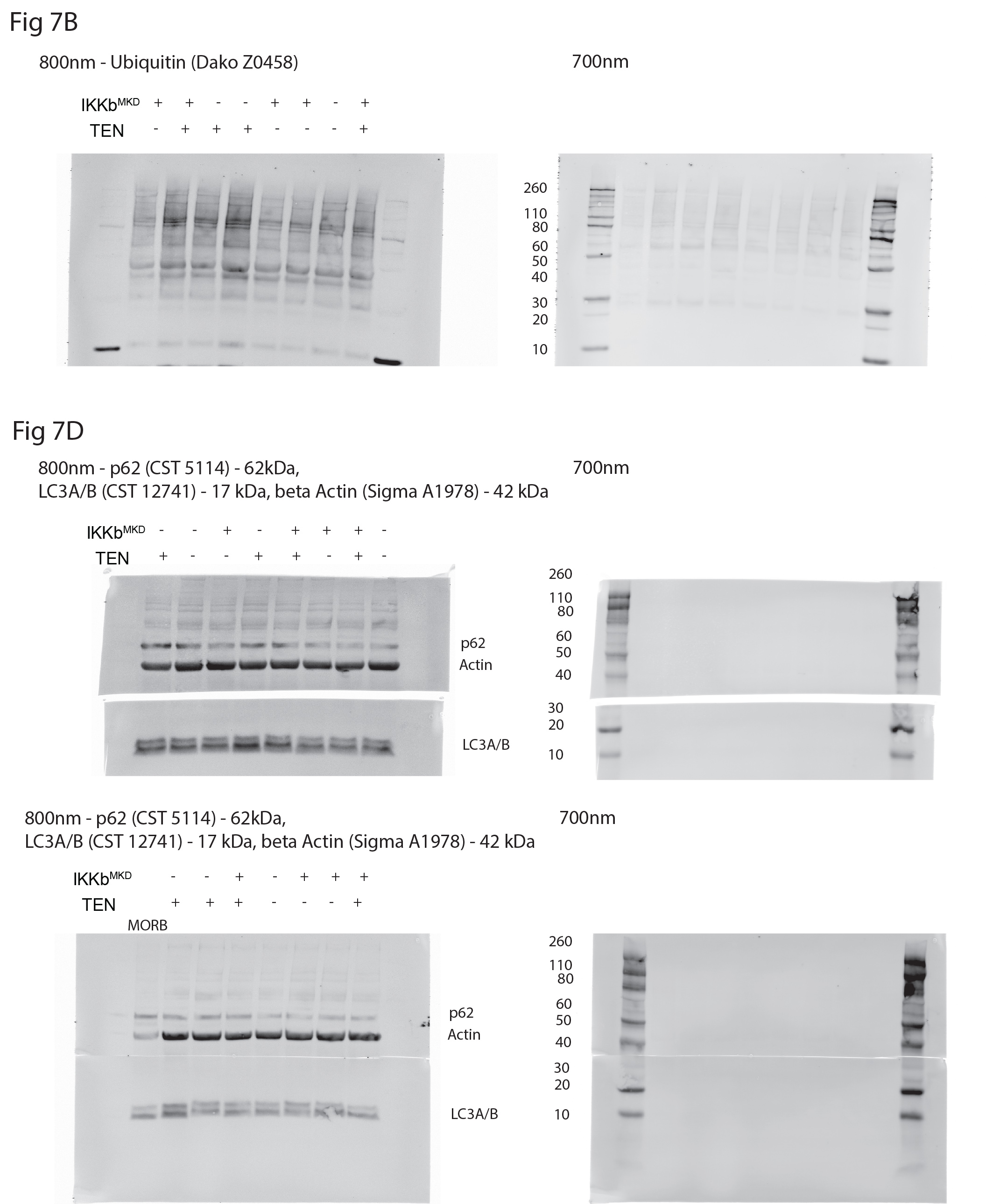

Supplement: Figure 7—source data 2. [file elife-82016-fig7-data2.zip › Fig 7 - source data 2/Fig7_Blots.jpg]

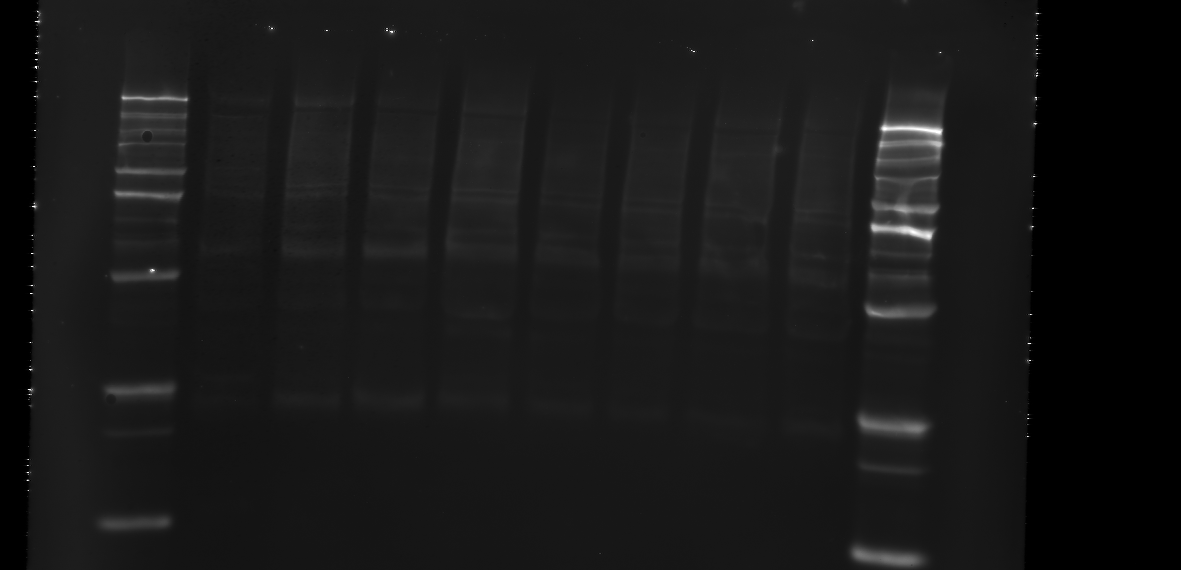

Supplement: Figure 7—source data 2. [file elife-82016-fig7-data2.zip › Fig 7 - source data 2/Fig 7B_Ubiquitin_700.TIF]

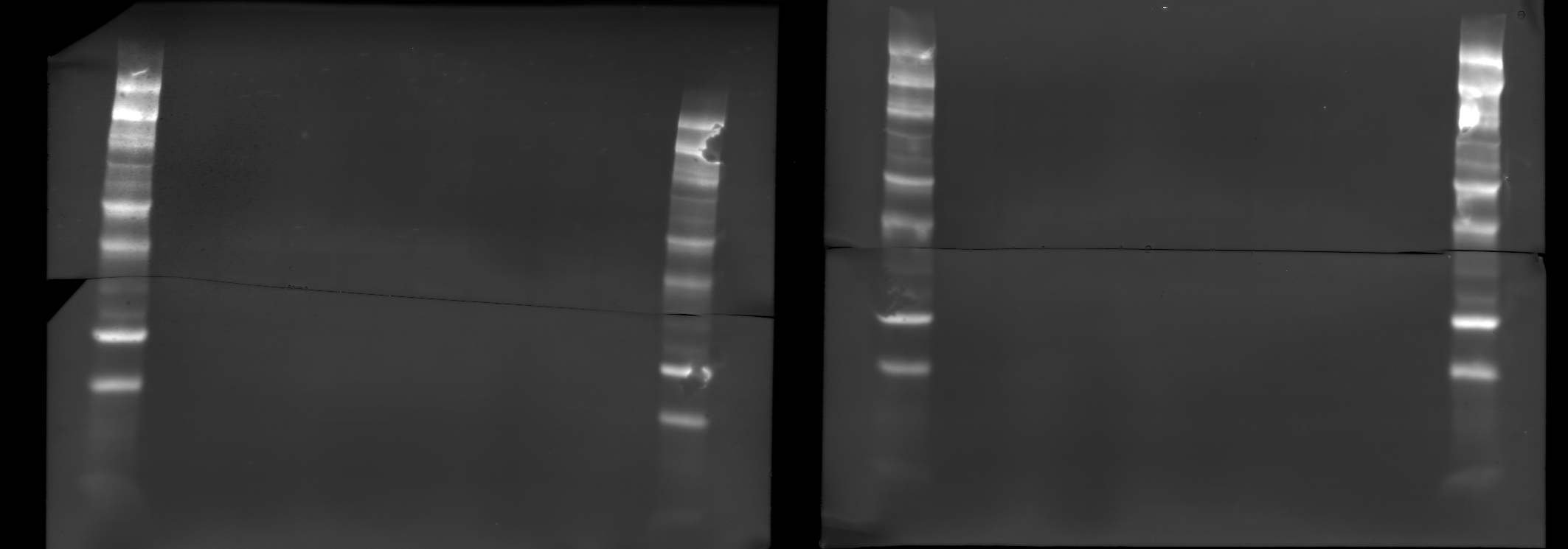

Supplement: Figure 7—source data 2. [file elife-82016-fig7-data2.zip › Fig 7 - source data 2/Fig7D2_p62_LC3_Actin_700.TIF]

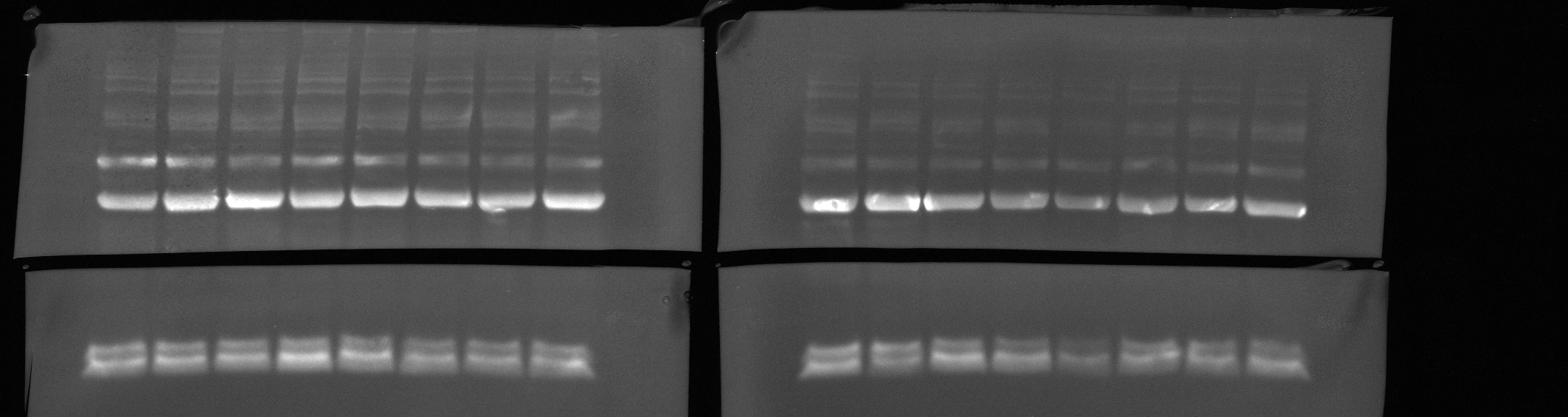

Supplement: Figure 7—source data 2. [file elife-82016-fig7-data2.zip › Fig 7 - source data 2/Fig7D_p62_LC3_Actin_800.TIF]

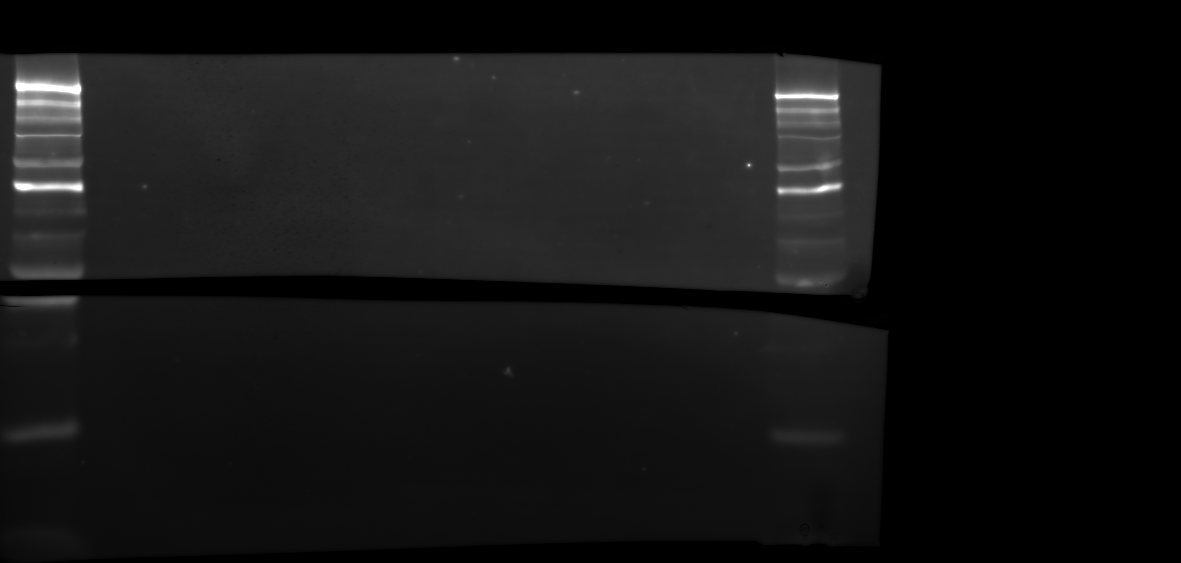

Supplement: Figure 7—figure supplement 1—source data 2. [file elife-82016-fig7-figsupp1-data2.zip › Fig 7S1 - source data 2/Fig7S1B_pAkt_700.TIF]

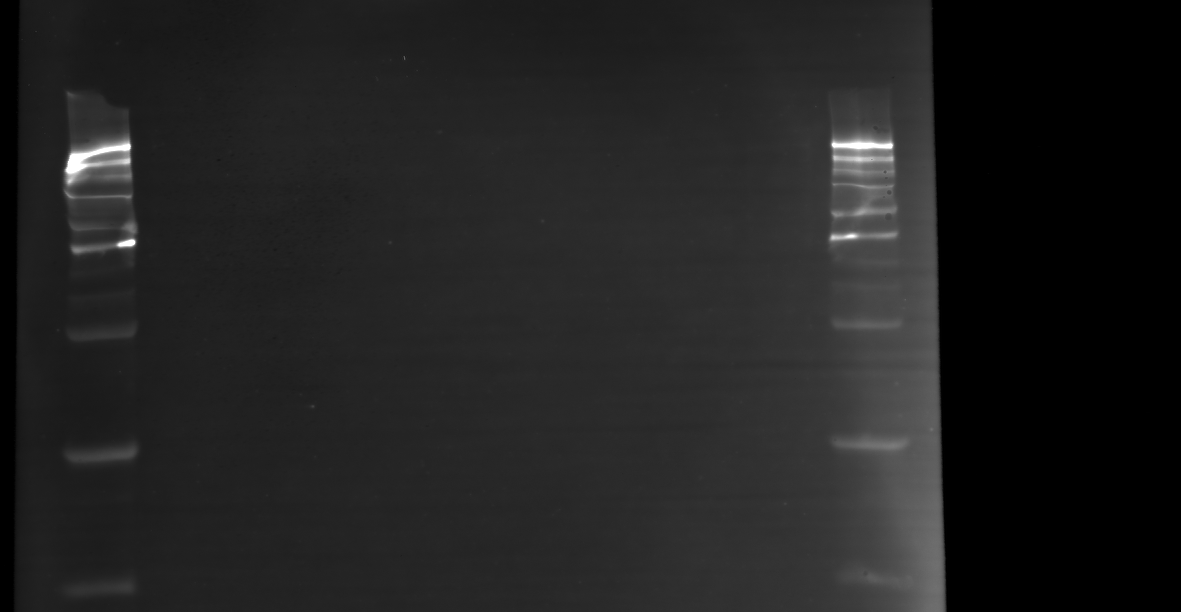

Supplement: Figure 7—figure supplement 1—source data 2. [file elife-82016-fig7-figsupp1-data2.zip › Fig 7S1 - source data 2/Fig7S1A_pmTor_pAkt_pS6_700.TIF]

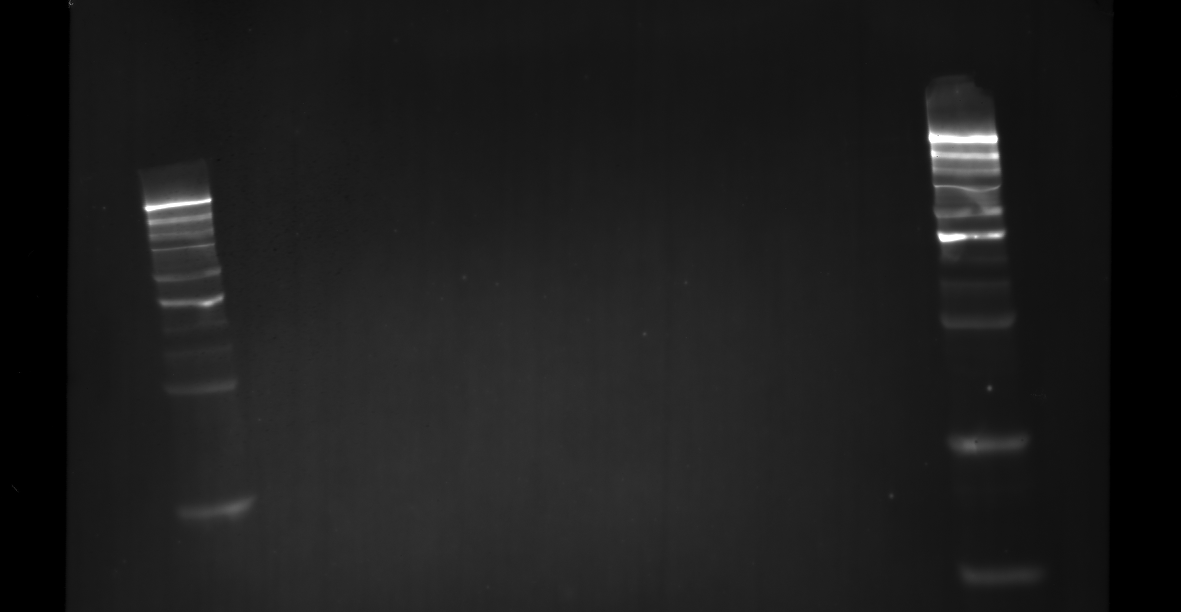

Supplement: Figure 7—figure supplement 1—source data 2. [file elife-82016-fig7-figsupp1-data2.zip › Fig 7S1 - source data 2/Fig7S1B_Akt_mTor_700.TIF]

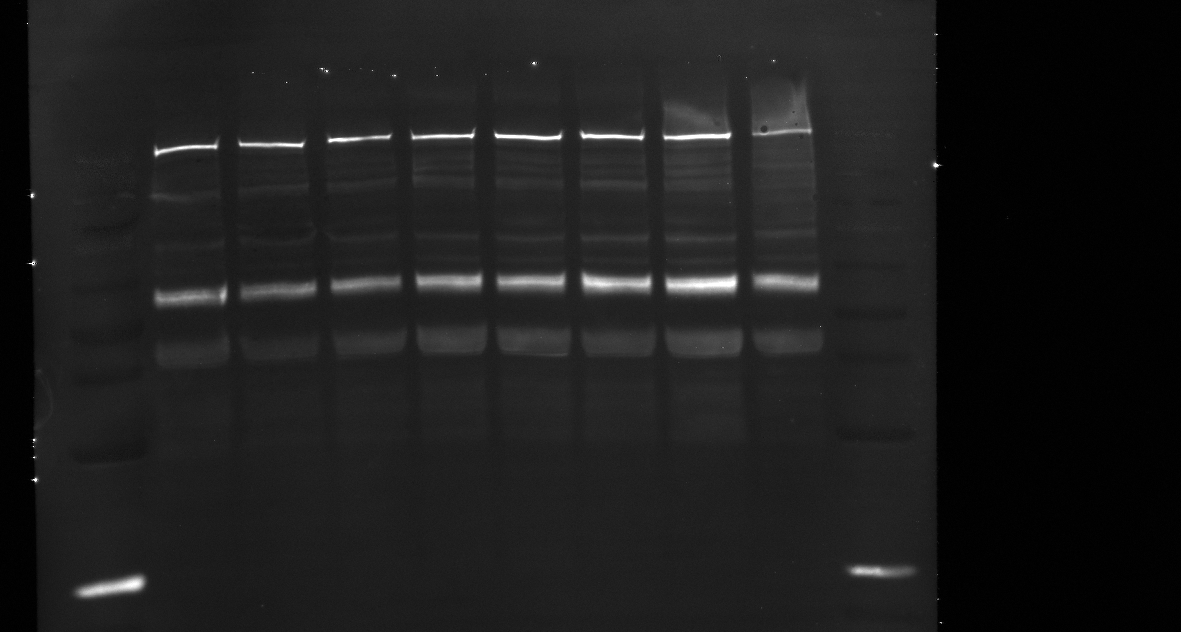

Supplement: Figure 7—figure supplement 1—source data 2. [file elife-82016-fig7-figsupp1-data2.zip › Fig 7S1 - source data 2/Fig7S1A_Akt_mTor_800.TIF]

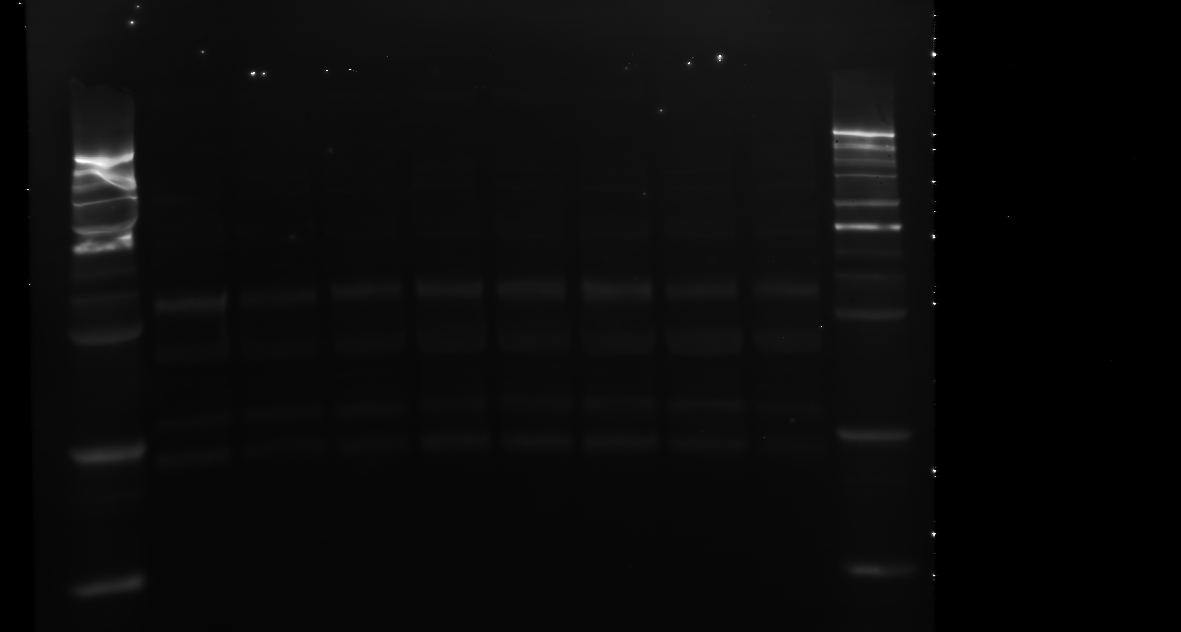

Supplement: Figure 7—figure supplement 1—source data 2. [file elife-82016-fig7-figsupp1-data2.zip › Fig 7S1 - source data 2/Fig7S1A_S6_700.TIF]

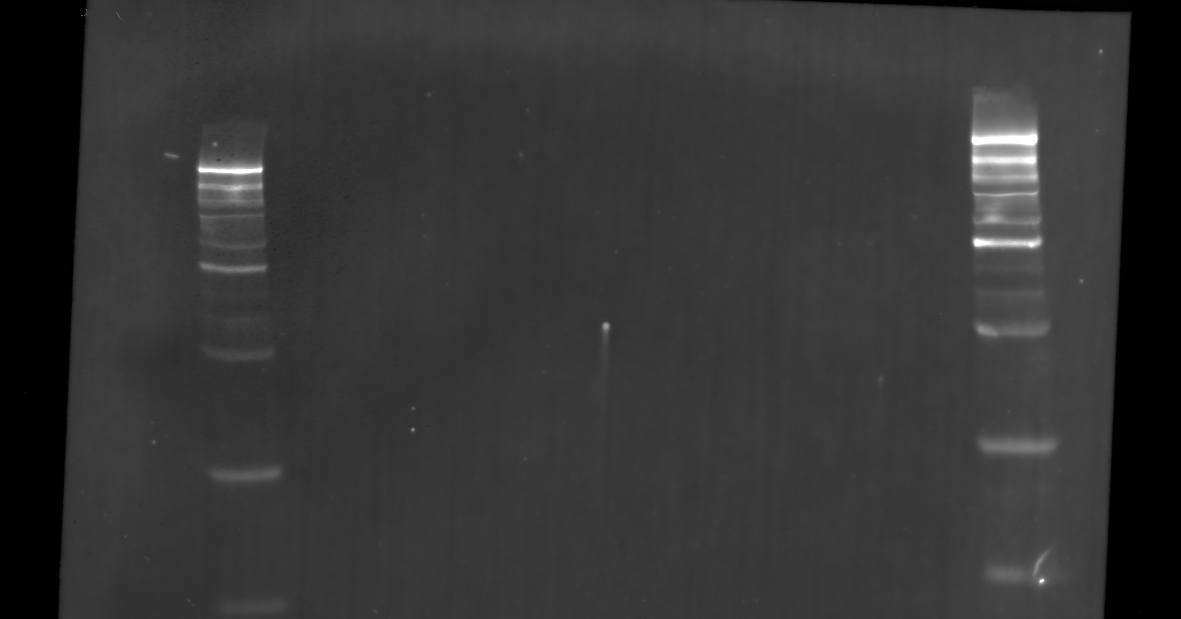

Supplement: Figure 7—figure supplement 1—source data 2. [file elife-82016-fig7-figsupp1-data2.zip › Fig 7S1 - source data 2/Fig7S1B_pmTor_700.TIF]

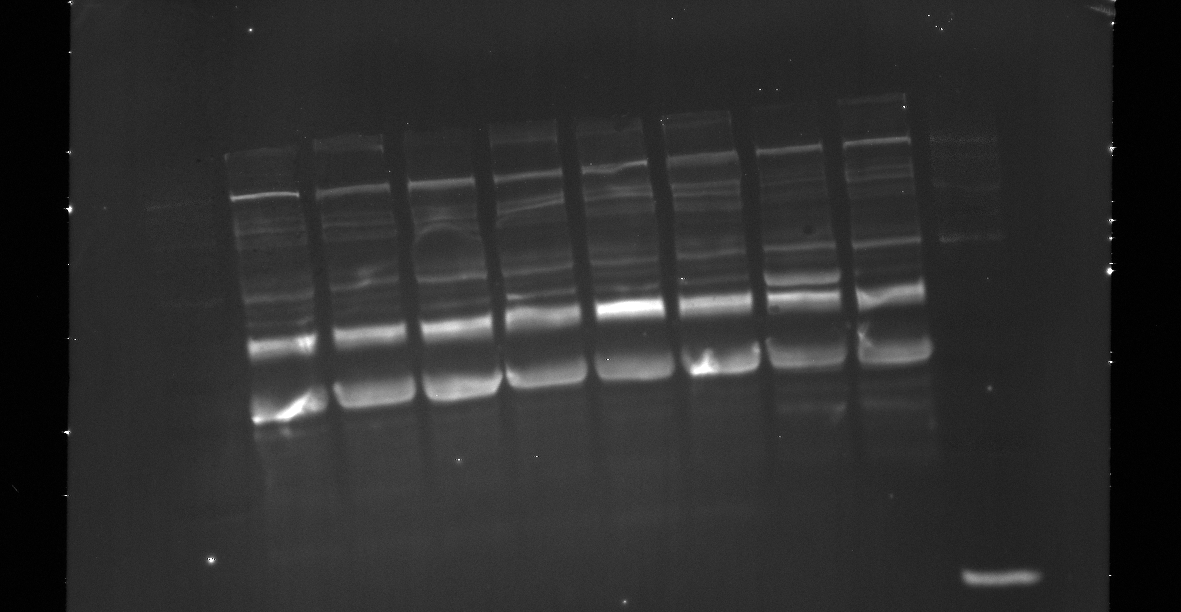

Supplement: Figure 7—figure supplement 1—source data 2. [file elife-82016-fig7-figsupp1-data2.zip › Fig 7S1 - source data 2/Fig7S1B_Akt_mTor_800.TIF]

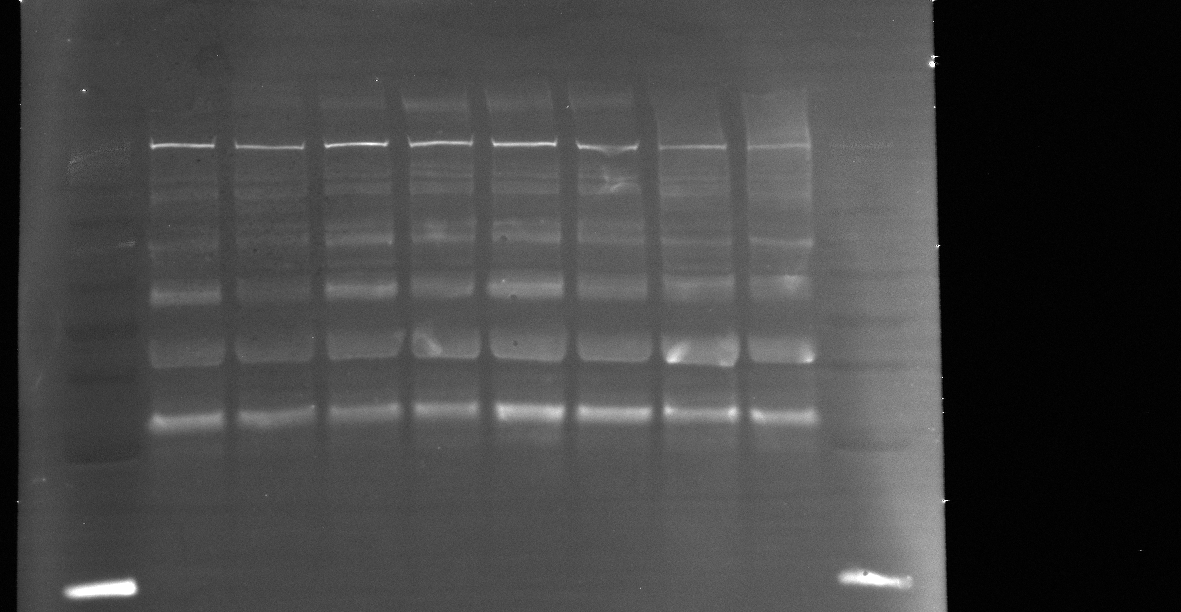

Supplement: Figure 7—figure supplement 1—source data 2. [file elife-82016-fig7-figsupp1-data2.zip › Fig 7S1 - source data 2/Fig7S1A_pmTor_pAkt_pS6_800.TIF]

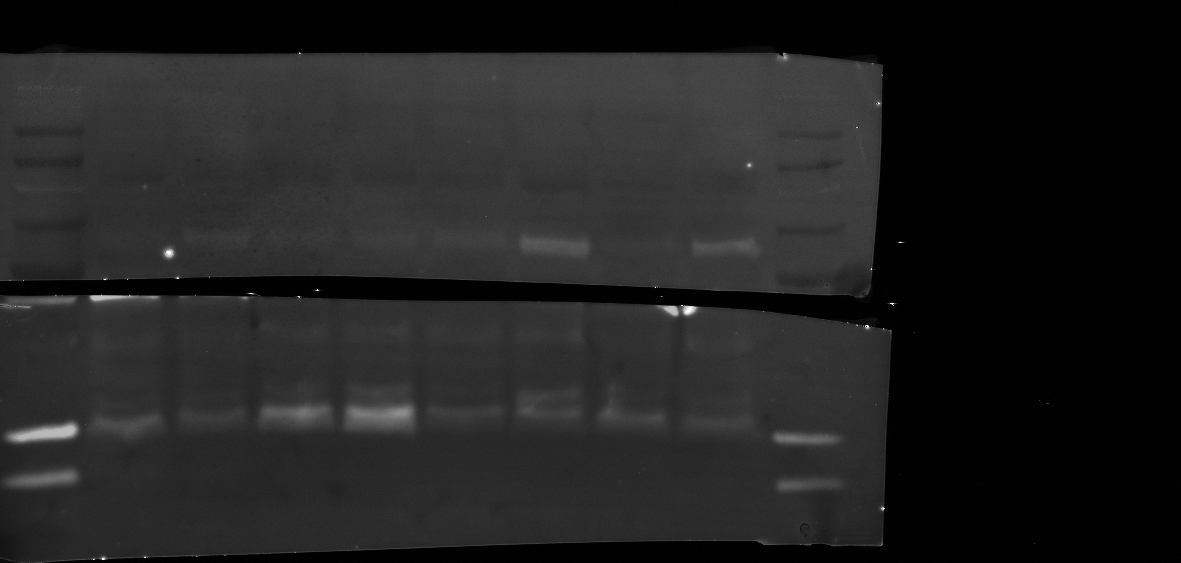

Supplement: Figure 7—figure supplement 1—source data 2. [file elife-82016-fig7-figsupp1-data2.zip › Fig 7S1 - source data 2/Fig7S1B_pAkt_800.TIF]

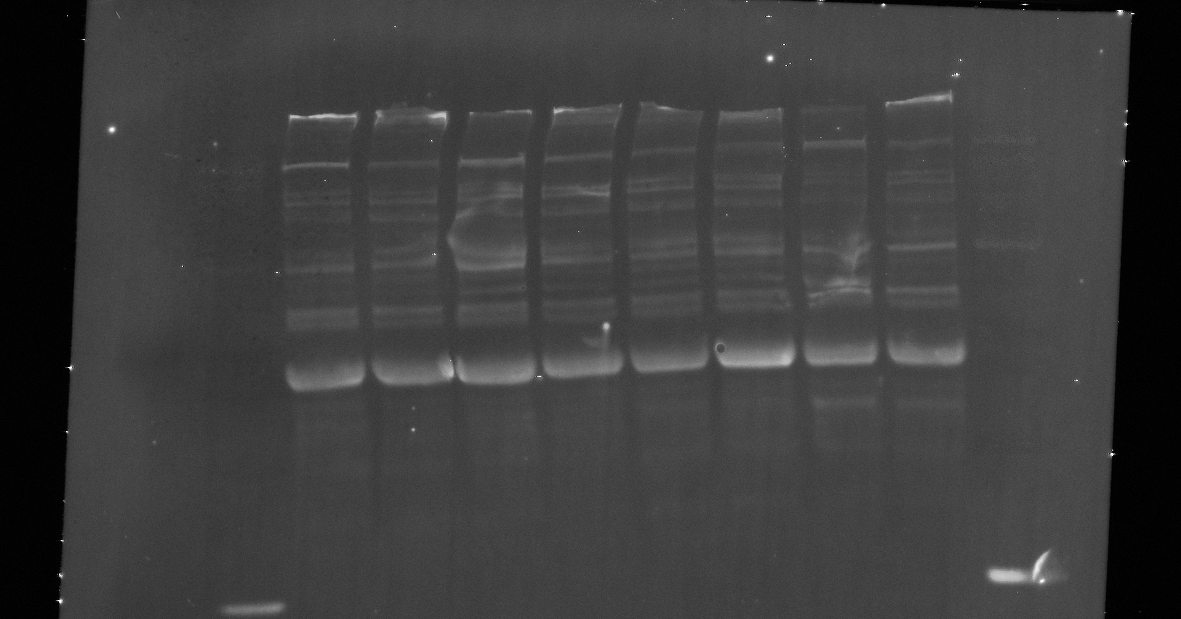

Supplement: Figure 7—figure supplement 1—source data 2. [file elife-82016-fig7-figsupp1-data2.zip › Fig 7S1 - source data 2/Fig7S1B_pmTor_800.TIF]

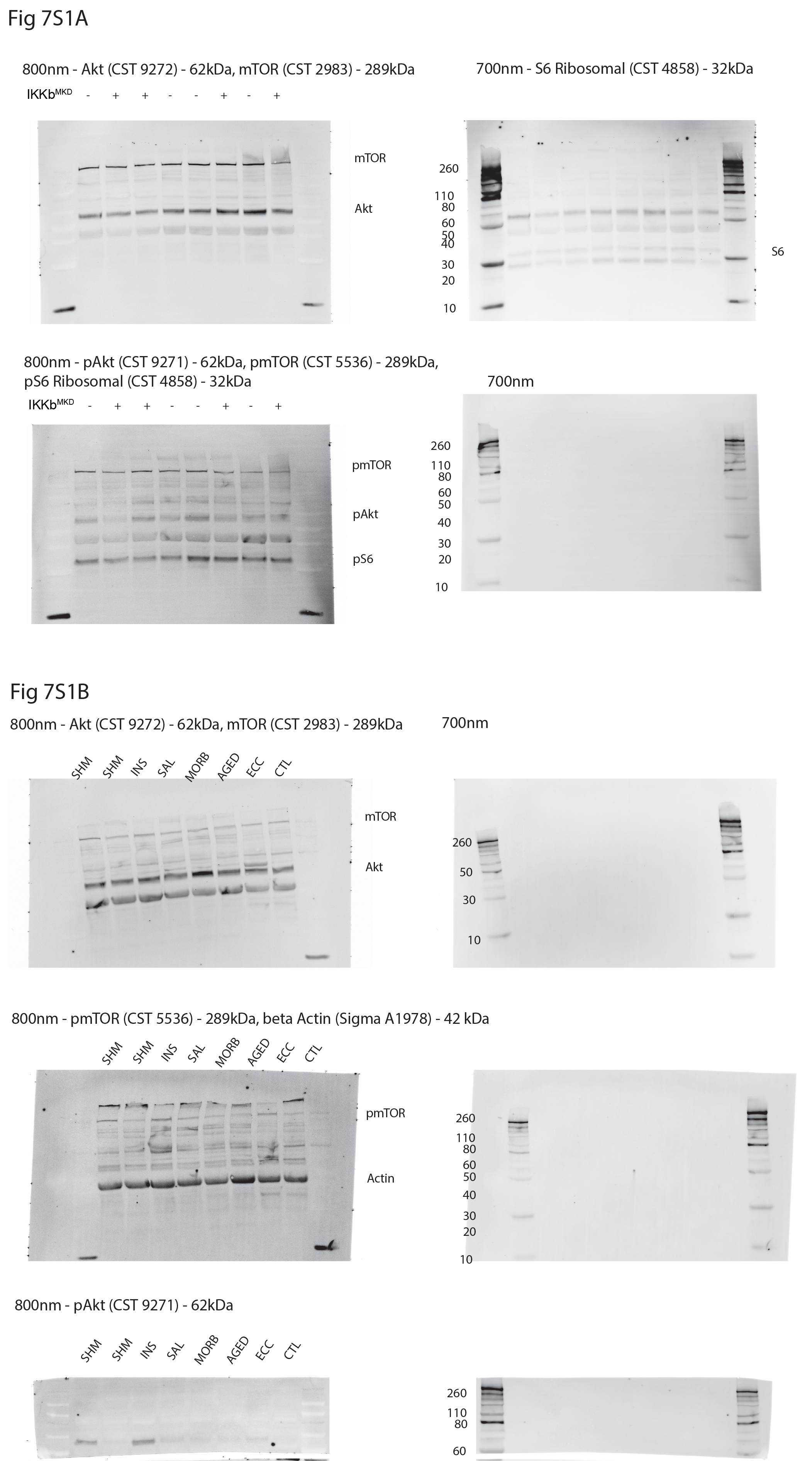

Supplement: Figure 7—figure supplement 1—source data 2. [file elife-82016-fig7-figsupp1-data2.zip › Fig 7S1 - source data 2/Fig7S1_Blots.jpg]

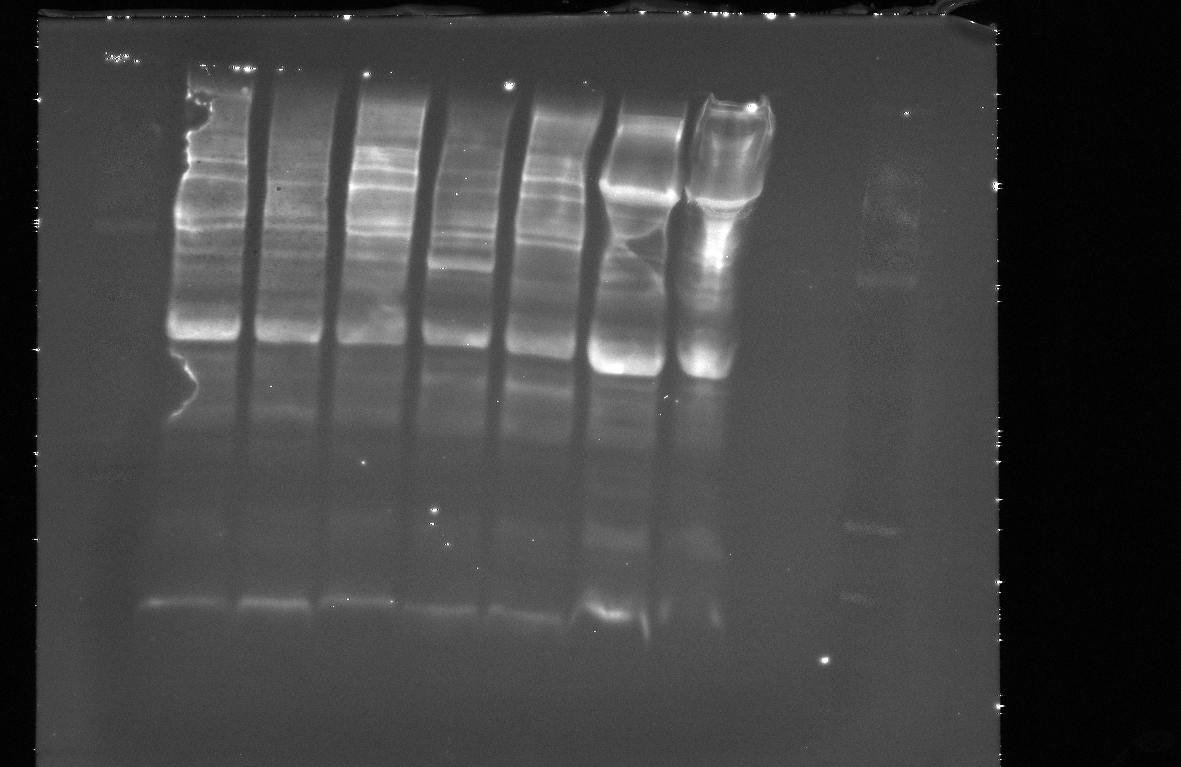

Supplement: Figure 7—figure supplement 2—source data 2. [file elife-82016-fig7-figsupp2-data2.zip › Fig 7S2 - source data 2/Fig 7S2B_ubiquitin_800.TIF]

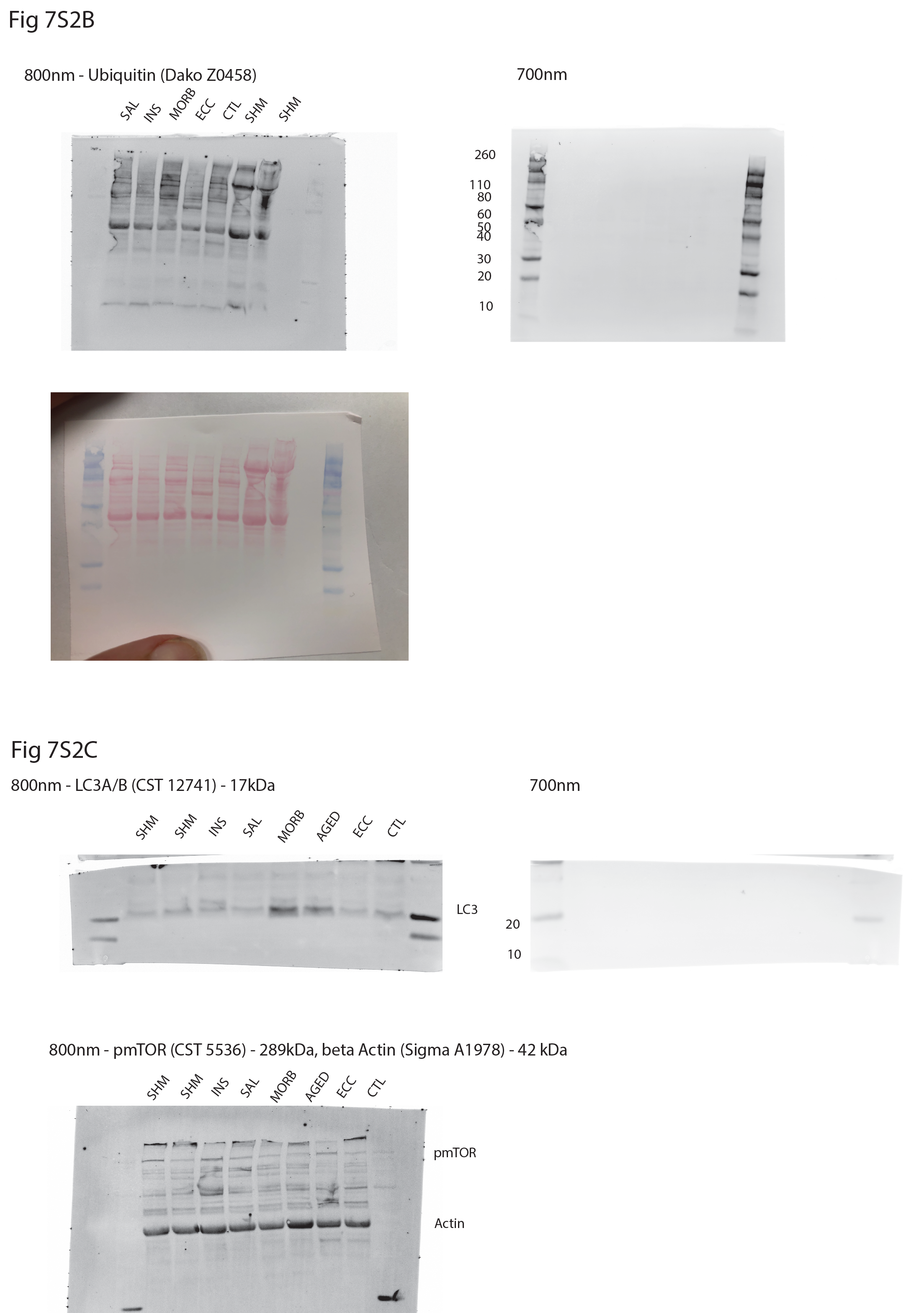

Supplement: Figure 7—figure supplement 2—source data 2. [file elife-82016-fig7-figsupp2-data2.zip › Fig 7S2 - source data 2/Fig7S2_Blots.tif]

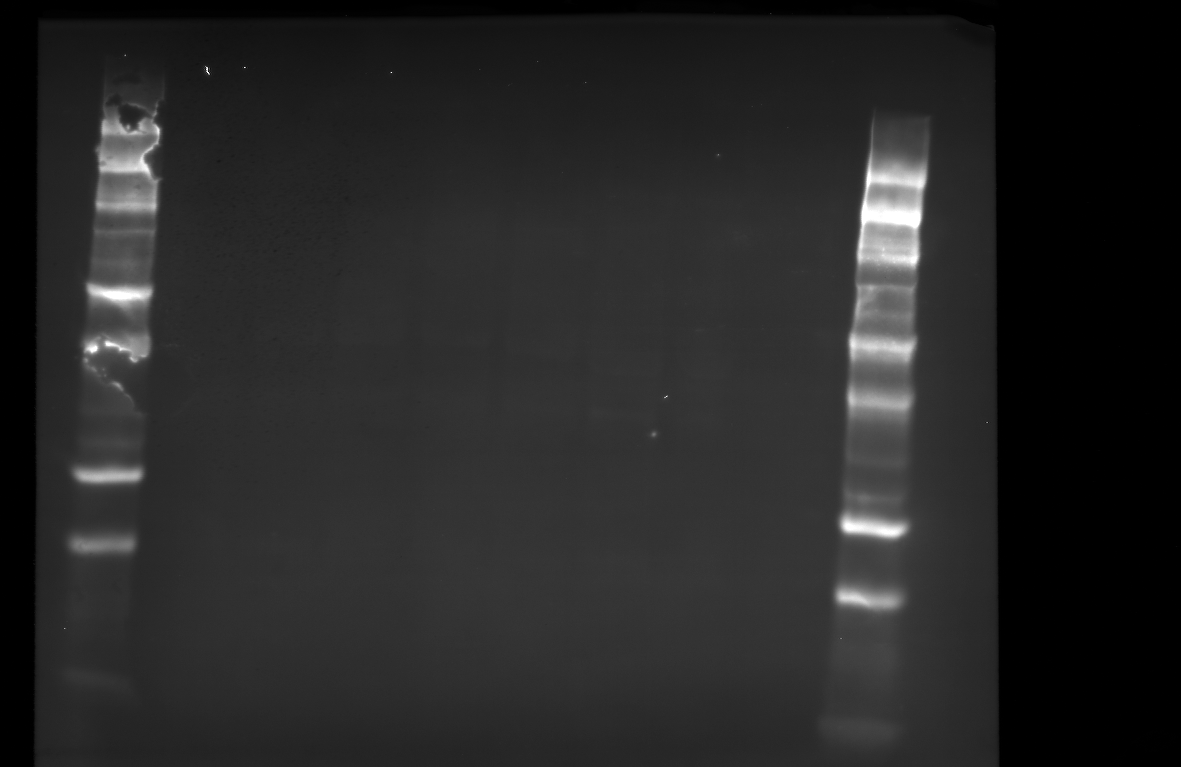

Supplement: Figure 7—figure supplement 2—source data 2. [file elife-82016-fig7-figsupp2-data2.zip › Fig 7S2 - source data 2/Fig 7S2B_ubiquitin_700.TIF]
